# Axitinib, cabozantinib, or everolimus in the treatment of prior sunitinib-treated patients with metastatic renal cell carcinoma: results of matching-adjusted indirect comparison analyses

**DOI:** 10.1186/s12885-018-5157-0

**Published:** 2018-12-19

**Authors:** Irina Proskorovsky, Agnes Benedict, Sylvie Negrier, Danielle Bargo, Rickard Sandin, Krishnan Ramaswamy, Jigar Desai, Joseph C. Cappelleri, James Larkin

**Affiliations:** 1 Evidera, 7575 Trans-Canada Highway, Suite 404, Montreal, Quebec H4R 1V6 Canada; 2 Evidera, Budapest, Hungary; 3 University of Lyon, Centre Léon Bérard, Lyon, France; 4 Pfizer Inc, New York, NY USA; 5 Pfizer AB, Vetenskapsvägen 10, 191 90 Sollentuna, Sweden; 6 Royal Marsden NHS Foundation Trust, London, UK

**Keywords:** Axitinib, Cabozantinib, Everolimus, Indirect comparison, Matching-adjusted comparison, mRCC, Prior sunitinib-treated patients

## Abstract

**Background:**

In the absence of head-to-head trials comparing axitinib with cabozantinib or everolimus, the aim of this study was to conduct an indirect comparison of their relative efficacy in patients with metastatic renal cell carcinoma (mRCC), using data from the AXIS and METEOR trials.

**Methods:**

Progression-free survival (PFS) and overall survival (OS) in prior sunitinib-treated patients with mRCC were compared by conducting matching-adjusted indirect comparison (MAIC) analyses, including base-case and sensitivity analyses. Individual patient-level data from prior sunitinib-treated patients who received axitinib in AXIS were weighted to match published baseline characteristics of prior sunitinib-treated patients who received either cabozantinib or everolimus in METEOR.

**Results:**

There was no statistically significant difference in PFS (aHR [adjusted hazard ratio] = 1.15 [CI: 0.82–1.63]) and OS (aHR = 1.00 [CI: 0.69–1.46]) between axitinib versus cabozantinib in the base-case analysis. In the sensitivity analysis, PFS (aHR = 1.39 [CI: 1.00–1.92]) and OS (aHR = 1.35 [CI: 0.95–1.92]) were shorter for axitinib compared with cabozantinib; however, the OS difference was not statistically significant. Axitinib was associated with significantly longer PFS compared with everolimus in the base-case (aHR = 0.53 [CI: 0.36–0.80]) and sensitivity analyses (aHR = 0.63 [CI: 0.45–0.88]), respectively. Results suggested an OS benefit for axitinib versus everolimus in base-case analyses (aHR = 0.63 [CI: 0.42–0.96]); however, the difference in OS in the sensitivity analysis was not statistically significant (aHR = 0.84 [CI: 0.59–1.18]).

**Conclusions:**

MAIC analyses suggest PFS and OS for axitinib and cabozantinib are dependent on the Memorial Sloan Kettering Cancer Center definition used; in the base-case analysis, there was no significant difference in PFS and OS between axitinib and cabozantinib. In the sensitivity analysis, PFS in favour of cabozantinib was significant; however, the trend for prolonged OS with cabozantinib was not significant. For axitinib and everolimus, MAIC analyses indicate patients treated with axitinib may have an improved PFS and OS benefit when compared to everolimus. Disparities between the base-case and sensitivity analyses in this study underscore the importance of adjusting for the differences in baseline characteristics and that naïve indirect comparisons are not appropriate.

## Background

Each year, approximately 214,000 patients are diagnosed with renal cell carcinoma (RCC) worldwide [1]. In 25–30% of these patients, the disease is already at the metastatic stage at presentation [2], which may be explained, in part, by lack of early symptoms for metastatic RCC (mRCC). The 5-year survival rate by American Joint Committee on Cancer (AJCC) tumour, lymph nodes, and metastasis (TNM) staging is 8% for Stage IV RCC [3]. The treatment landscape for patients with mRCC has evolved substantially in the past decade with the introduction of targeted therapies, which has led to significant improvements in patient outcomes.

For patients with mRCC who progress on prior targeted therapy, mostly with sunitinib, treatment options available as second-line and/or subsequent systemic therapies include axitinib, everolimus, lenvatinib in combination with everolimus, cabozantinib, and nivolumab. With an increasing number of targeted agents being approved for the treatment of mRCC, data from comparative studies of these agents would help attending physicians and patients to make decisions based on individualized treatment algorithms. Due to the limited number of head-to-head clinical trials that directly evaluate these targeted agents as second-line and/or subsequent therapy for mRCC, an indirect treatment comparison analysis is needed. Axitinib was a well-established targeted agent in the second-line setting before other targeted therapies were introduced. Axitinib has been compared with sorafenib in the Axitinib as Second Line Therapy for Metastatic Renal Cell Carcinoma (AXIS) trial [4, 5]. Everolimus has been compared with cabozantinib in the Cabozantinib versus Everolimus in Advanced Renal Cell Carcinoma (METEOR) trial [6, 7], and with placebo in the Renal Cell Cancer Treatment with Oral RAD001 given Daily (RECORD-1) trial [8]. A network meta-analysis (NMA) is an indirect treatment comparison technique commonly used to estimate relative treatment effects based on published data from different trials. This technique relies on the availability of a connected network to contrast relative effects between treatments, and assumes homogeneity of trials included in the network. An NMA is not a suitable method to perform a comparison of axitinib with everolimus or cabozantinib, since there is no comparator that links axitinib to either of these two agents in a population of patients who received prior sunitinib. Furthermore, there are important differences in parameters, such as patient baseline characteristics, observed between the trials that are available to create a connected network. In such a case, an alternative indirect treatment comparison approach, such as a matching-adjusted indirect comparison (MAIC) [9], may be more appropriate.

The MAIC technique has been acknowledged in health technology assessments in oncology [10, 11] and applied to generate comparative evidence in several diseases [12–17]. Unlike a naïve indirect comparison that is based on the observed outcomes of two arms across different trials without adjustment for baseline differences (and therefore subject to confounding by both the observed and unobserved baseline differences between the trials), MAIC analyses compare treatments using information from compatible studies while adjusting for differences in the population characteristics across trials. Patient-level data from one trial (the index trial) are adjusted to the baseline characteristics of the comparator trial, thereby making it possible to compare outcomes across trials.

The aim of this study was to compare progression-free survival (PFS) and overall survival (OS) in prior sunitinib-treated patients who received axitinib in the AXIS trial [4] with prior sunitinib-treated patients who received either everolimus or cabozantinib in the METEOR trial [6, 7], using MAIC analyses.

## Methods

### Study populations and treatments

The AXIS trial was a randomised, phase 3 trial of axitinib compared with sorafenib, a multi-targeted tyrosine kinase inhibitor (TKI), in previously treated patients with mRCC [4]. The trial demonstrated improved PFS for axitinib versus sorafenib [4], although OS did not differ between the two treatments [5]. The randomised, phase 3 METEOR trial compared everolimus, a mammalian target of rapamycin inhibitor, with cabozantinib, another multi-targeted TKI, in mRCC patients who progressed on prior systemic therapy [6, 7, 18, 19]. The METEOR trial demonstrated improved PFS and OS with cabozantinib versus everolimus. Patient-level data from the AXIS trial (index trial) and published data from the METEOR trial were used for the current analyses.

In the AXIS trial, eligible patients had mRCC with a clear cell component, measurable disease by Response Evaluation Criteria in Solid Tumours (RECIST) v1.0, Eastern Cooperative Oncology Group performance status (ECOG PS) 0 or 1, and progressed as assessed by investigators following one prior systemic first-line therapy with sunitinib, bevacizumab plus interferon-alpha, temsirolimus, or cytokines [4]. Patients in the axitinib arm received a starting dose of axitinib 5 mg orally twice daily; those who tolerated the starting dose per the predefined set of criteria were allowed to have their dose increased to 7 mg twice daily, up to a maximum of 10 mg twice daily [4]. For these analyses, patient-level data of the prior sunitinib-treated patient subgroup were used.

Eligible patients in the METEOR trial had mRCC with a clear cell component, measurable disease by RECIST v1.1, and Karnofsky performance status (KPS) score ≥ 70%. Patients must have received previous therapy with at least one vascular endothelial growth factor receptor (VEGFR)-targeting TKI (no limit to the number of prior therapies for RCC, including inhibitors of programmed cell death-1 [PD-1] or programmed cell death ligand-1 [PD-L1]). They must also have had radiographic progression during therapy or within 6 months after the last dose of VEGFR inhibitors [6, 18, 19]. Patients were randomly assigned to receive an oral daily dose of either 10 mg everolimus or 60 mg cabozantinib [6, 18, 19]. As with the index trial, only the data from the prior sunitinib-treated patient subgroups were used for these analyses.

### Outcomes

The primary outcome in both trials was PFS; it was defined as the time from randomisation to either first documentation of RECIST-defined disease progression or death due to any cause, whichever came first. PFS was evaluated by independent review committee using the RECIST v1.0 (AXIS) or v1.1 (METEOR) criteria. A secondary outcome in both trials was OS, defined as the time from randomisation to death. Published METEOR data on PFS and OS were extracted using Engauge Digitizer, and patient-level data for each curve were generated using the methods described by Guyot et al. [20]. The accuracy of the derived patient-level data was checked by plotting the resulting Kaplan–Meier curves against the coordinates from the published graphs. Since the AXIS and METEOR trials were conducted at different times, with likely differences in treatment pathways and multiple treatment options available post-study, PFS was considered the primary outcome measure and OS the secondary outcome measure in the current analyses.

### Statistical analyses

#### Compatibility assessment

A compatibility assessment was performed to determine the feasibility of conducting MAIC analyses with the available data through a comparative review of the trial design, population profiles, and outcome measures of the AXIS and METEOR trials.

#### Matching-adjusted indirect comparison

Following the compatibility assessment, the MAIC was conducted as outlined by Signorovitch et al. [9]. Weights based on the MAIC were assigned to patients in the prior sunitinib-treated patient subgroup from the AXIS trial, to balance the differences in baseline characteristics compared with the prior sunitinib-treated patient subgroups in the METEOR trial. Baseline characteristics used for matching included: age, sex, histology of mRCC, ECOG PS or KPS, metastatic site (bone, lung, liver, lymph), previous radiotherapy, previous nephrectomy, and geographic region) and Memorial Sloan Kettering Cancer Center (MSKCC) scores. In the METEOR trial, MSKCC scores were calculated using KPS, as per the original definition by Motzer et al. [21]; ECOG PS was used in the AXIS trial, since KPS was not collected. Given the importance of MSKCC score in determining patient prognosis, and in the absence of an established method for mapping from ECOG PS to KPS, two sets of analyses were conducted to assess the robustness of the results to differences in MSKCC scores definitions between the AXIS and METEOR trials – base-case and sensitivity analyses. For the base-case analysis, all patient characteristics were used in matching, including the MSKCC score that was derived assuming ECOG PS 1 was a risk factor (MSKCC score as defined in the AXIS trial). Similarly, for the sensitivity analysis, the same patient characteristics as in the base-case analysis were used in matching, except that the MSKCC score in the AXIS trial was derived assuming ECOG PS 1 was not a risk factor.

A propensity-score logistic regression equation was used to reweight the data from the prior sunitinib-treated patients who were treated with axitinib in the AXIS trial, so that their aggregate characteristics matched exactly with those in the METEOR trial for all baseline characteristics that were available in both studies. Race was not included in the matching process as it was strongly correlated with geographic region. Time since diagnosis, number of metastases, and duration of the first TKI treatment were not included, as they were not reported for the subgroup of prior sunitinib-treated patients in the METEOR trial. Effective sample size (ESS) was derived as (∑*w*_*i*_)^2^/(∑*w*_*i*_^2^), where *w*_*i*_ represents weights for the *i*^th^ patient. A low ESS may indicate an irregular distribution of weights across patients, with a large fraction of patients with very small weights.

Obtained weights were applied to derive adjusted PFS and OS curves for axitinib using a Kaplan–Meier approach. The adjusted survival curves represented the expected survival outcomes of axitinib in the METEOR-like population, which were then compared graphically with observed curves for prior sunitinib-treated patients who were treated with either everolimus or cabozantinib in the METEOR trial. The relative effect of axitinib versus everolimus, and of axitinib versus cabozantinib was calculated as adjusted hazard ratio (aHR) with 95% confidence interval (CI) in the MAIC. The aHRs were obtained using a Cox proportional hazard regression analysis based on the weighted patient-level data in the AXIS trial and derived patient-level data for PFS and OS in the METEOR trial; the 95% CI for the aHR estimate took the ESS into account. Median PFS, median OS, and 95% CIs were estimated based on the weighted Kaplan–Meier analysis.

## Results

### Compatibility assessment

#### Study designs

The AXIS and METEOR trials were generally similar in design. Both were phase 3 trials with comparable inclusion/exclusion criteria and geographic coverage (most patients enrolled from the United States and Europe). In addition, both trials required disease progression on prior treatment before enrolling, and had similar definitions of PFS and OS.

However, there were important differences in study design between the two trials. First, MSKCC score in the AXIS trial was calculated using ECOG PS, whereas KPS was used for this calculation in the METEOR trial. Secondly, the AXIS trial enrolled patients who progressed after one prior first-line systemic therapy, including sunitinib, bevacizumab plus interferon-alpha, temsirolimus, or cytokines; the METEOR trial enrolled patients who received prior therapy with at least one VEGFR-targeted TKI and experienced progression, resulting in 30% of the patients having ≥2 prior VEGFR therapies. For the current analysis, only prior sunitinib-treated patient subgroups from both trials were used. Finally, PFS assessment schedules differed between the trials (AXIS: after 6 and 12 weeks of therapy, and every 8 weeks thereafter; METEOR: every 8 weeks for the first 12 months, and every 12 weeks thereafter).

#### Patient characteristics

Prior sunitinib-treated patients who were subsequently treated with axitinib in the AXIS trial, and those treated with everolimus or cabozantinib in the METEOR trial, were generally similar in terms of age, sex, geographic region, previous nephrectomy, and histology (Table 1). Prior to adjusting for differences in baseline characteristics, a higher proportion of patients with lung metastases, and a lower proportion with ECOG PS 0 and prior radiotherapy were included in the AXIS trial compared with the prior sunitinib-treated subgroup in the METEOR trial. In the AXIS trial, 52% of prior sunitinib-treated patients who were then treated with axitinib had an ECOG PS 0, compared with 66 and 70% of prior sunitinib-treated patients who were treated with everolimus and cabozantinib, respectively, in the METEOR trial. Furthermore, a lower proportion of patients were in the favourable risk group in the AXIS trial (20% in the base-case analysis, 33% in the sensitivity analysis) versus the everolimus arm (45%) or cabozantinib arm (41%), regardless of MSKCC score definition, in the METEOR trial (Table 1).

**Table 1:** Baseline demographics and characteristics before and after matching in prior sunitinib-treated patients

Trial	AXIS	AXIS	METEOR	AXIS	METEOR
Arm	Axitinib, before matching (*N* = 194)	Axitinib, after matching vs. cabozantinib (ESS = 104/114)	Cabozantinib (*N* = 135)	Axitinib, after matching vs. everolimus (ESS = 61/95)	Everolimus (*N* = 132)
Sex, %					
Male	74	79	79	72	72
Female	26	21	21	28	28
Median age, years	62	62	62	62	62
Geographic regions, %					
Europe	51	53	53	50	50
North America	29	33	33	33	33
Asia	15	13	13	17	17
Other	5	< 1	< 1	1	1
ECOG PS or KPS, %					
0 (KPS 90–100)	52	70	70	66	66
1 (KPS 70–80)	48	30	30	34	34
MSKCC in the base-case analysis, %					
Favourable	20	41	41	45	45
Intermediate	42	47	47	44	44
Poor	34	13	13	11	11
NR	4	0	0	0	0
MSKCC in the sensitivity analysis, %					
Favourable	33	41	41	45	45
Intermediate	58	47	47	44	44
Poor	5	13	13	11	11
NR	4	0	0	0	0
Histology, %					
Clear cell or clear cell component	98	100	100	100	100
Metastatic site, %					
Lung	73	59	59	67	67
Bone	30	20	20	42	42
Liver	33	32	32	17	17
Prior nephrectomy, %	88	86	86	85	85
Prior radiotherapy, %	23	29	29	31	31

*ECOG PS* Eastern Cooperative Oncology Group performance status, *ESS* effective sample size, *KPS* Karnofsky performance score, *MSKCC* Memorial Sloan Kettering Cancer Center, *NR* not reported

More patients in the AXIS trial received post-study subsequent therapies than those in the METEOR trial, and the composition of post-study therapies were different between the trials (Table 2). A smaller percentage of patients in the METEOR trial had access to immune-oncology therapies, such as PD-1/PD-L1 inhibitors.

**Table 2:** Subsequent therapy use in AXIS and METEOR

Trial	AXIS	METEOR	METEOR
Arm	Axitinib, prior sunitinib-treated patients (*N* = 194)	Cabozantinib, prior sunitinib-treated patients (*N* = 135)	Everolimus, prior sunitinib-treated patients (*N* = 132)
Any systemic therapy, %	60	48	55
Axitinib	0	18	39
Pazopanib	5	3	7
Sunitinib	7	2	3
Sorafenib	20	2	7
Cabozantinib	0	0	2
Everolimus	43	25	4
Bevacizumab	8	2	2
Anti-PD-1/PD-L1	0	2	4

*PD-1* programmed cell death-1, *PD-L1* programmed cell death ligand-1

Patients may have received more than one subsequent therapy

### Efficacy

#### Progression-free survival

##### Naïve indirect comparison

The naïve indirect comparative analysis before adjustments showed a shorter PFS for axitinib than cabozantinib: median PFS was 4.8 months (95% CI 4.5–6.5) for axitinib versus 9.1 months (95% CI 6.4–9.4) for cabozantinib (Table 3). For axitinib compared with everolimus, PFS was longer with median PFS 4.8 months (95% CI 4.5–6.5) for axitinib versus 3.7 months (95% CI 3.5–4.4) for everolimus (Table 3).

**Table 3:** Progression-free survival – naïve and MAIC (base-case and sensitivity) analyses

	Naïve	Base-case analysis	Sensitivity analysis
Axitinib (AXIS) vs. cabozantinib (METEOR)			
Axitinib, median (95% CI) PFS, months	4.8 (4.5–6.5)	6.5 (4.7–10.4)	4.8 (4.2–6.7)
Cabozantinib, median (95% CI) PFS, months	9.1 (6.4–9.4)	9.1 (6.4–9.4)	9.1 (6.4–9.4)
aHR (95% CI)	–	1.15 (0.82–1.63)	1.39 (1.00–1.92)
Axitinib (AXIS) vs. everolimus (METEOR)			
Axitinib, median (95% CI) PFS, months	4.8 (4.5–6.5)	6.5 (4.7–11.0)	6.5 (4.6–7.8)
Everolimus, median (95% CI) PFS, months	3.7 (3.5–4.4)	3.7 (3.5–4.4)	3.7 (3.5–4.4)
aHR (95% CI)	–	0.53 (0.36–0.80)	0.63 (0.45–0.88)

*aHR* adjusted hazard ratio, *CI* confidence interval, *MAIC* matching-adjusted indirect comparison, *MSKCC* Memorial Sloan Kettering Cancer Center, *PFS* progression-free survival

##### Matching-adjusted indirect comparison

After matching, baseline characteristics were balanced between prior sunitinib-treated patients who received axitinib in the AXIS trial and those who received either cabozantinib or everolimus in the METEOR trial (Table 1). The ESS for axitinib was 104 (base-case) and 114 (sensitivity) patients when matched to cabozantinib-treated patients in the METEOR trial, and 61 (base-case) and 95 (sensitivity) patients when matched to everolimus-treated patients.

**Fig. 1:**
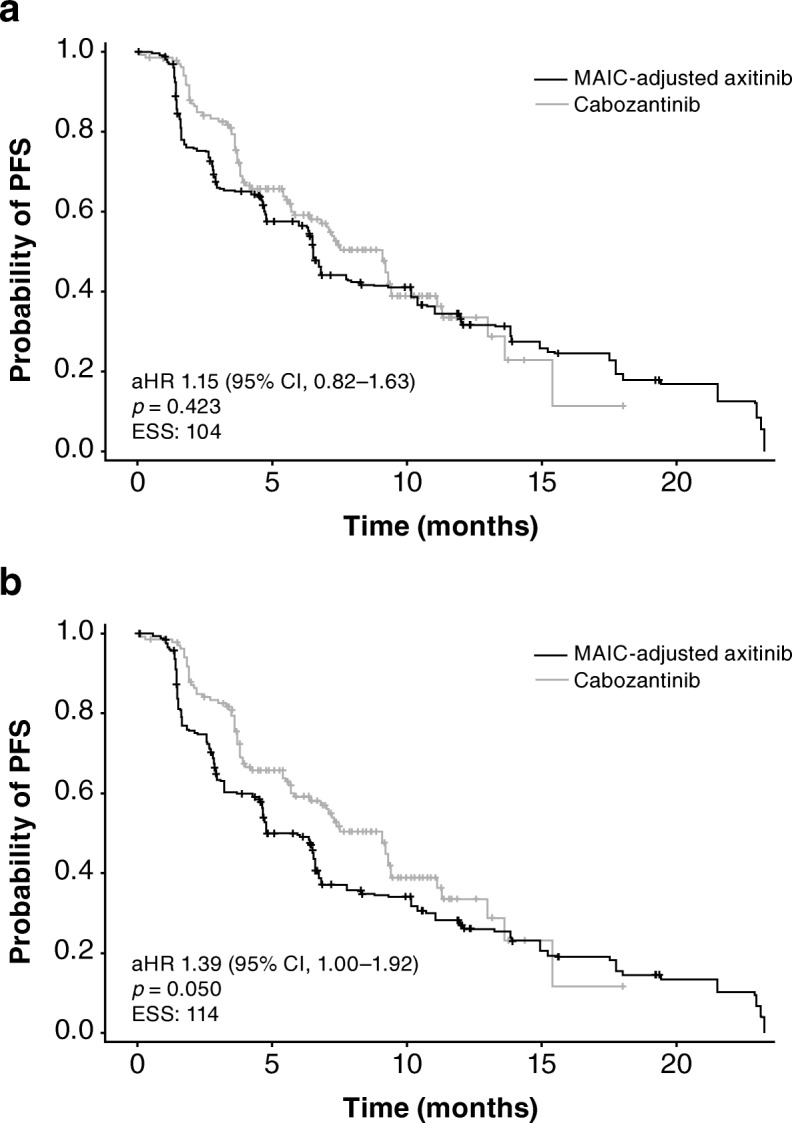
Kaplan–Meier curves for progression-free survival for axitinib versus cabozantinib: (**a**) base-case analysis; (**b**) sensitivity analysis. aHR, adjusted hazard ratio; CI, confidence interval; ESS, effective sample size; MAIC, matching-adjusted indirect treatment comparison; PFS, progression-free survival

**Fig. 2:**
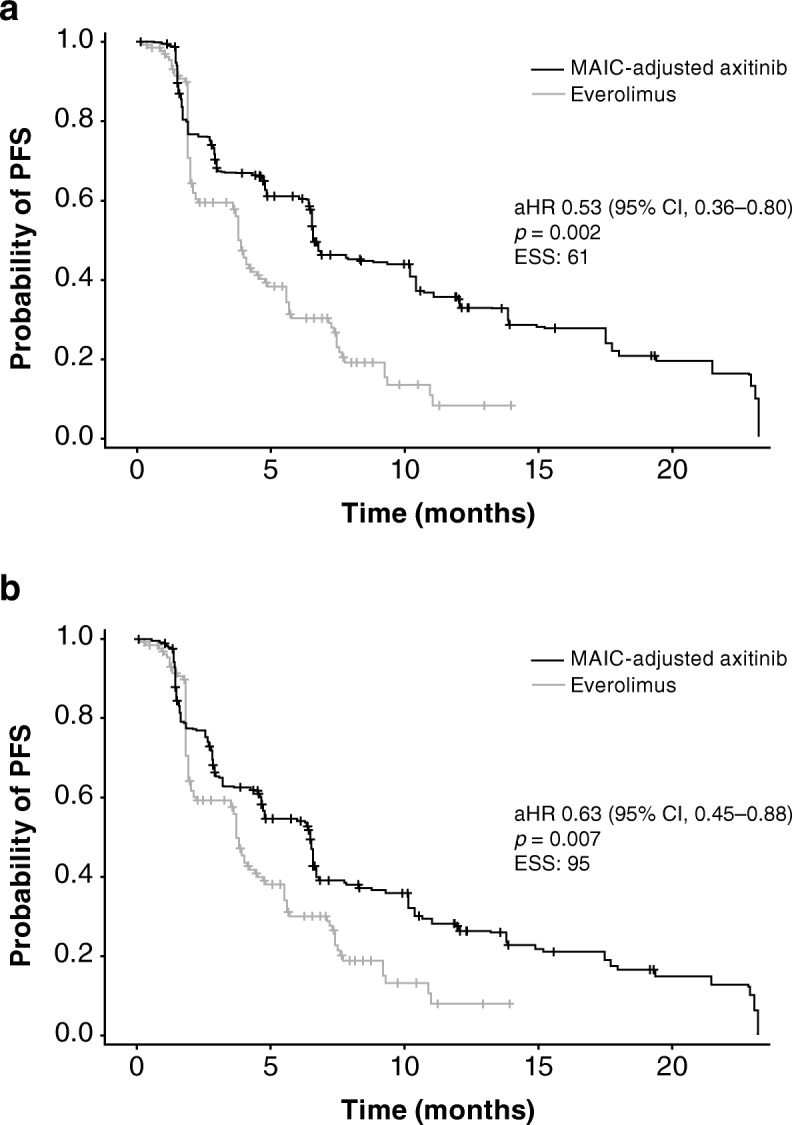
Kaplan–Meier curves for progression-free survival for axitinib versus everolimus: (**a**) base-case analysis; (**b**) sensitivity analysis. aHR, adjusted hazard ratio; CI, confidence interval; ESS, effective sample size; MAIC, matching-adjusted indirect treatment comparison; PFS, progression-free survival

In the base-case analysis, there was no statistically significant difference in PFS between axitinib and cabozantinib: aHR was 1.15 (95% CI 0.82–1.63; *p* = 0.423); and estimated median PFS was 6.5 months (95% CI 4.7–10.4) for axitinib versus 9.1 months (95% CI 6.4–9.4) for cabozantinib (Table 3; Fig. 1). PFS was longer for axitinib than everolimus: aHR was 0.53 (95% CI 0.36–0.80; *p* = 0.002); and estimated median PFS was 6.5 months (95% CI 4.7–11.0) for axitinib versus 3.7 months (95% CI 3.5–4.4) for everolimus (Table 3; Fig. 2).

In the sensitivity analysis, PFS was shorter for axitinib compared with cabozantinib: aHR was 1.39 (95% CI 1.00–1.92; *p* = 0.050); and estimated median (95% CI) PFS was 4.8 months (95% CI 4.2–6.7) for axitinib versus 9.1 months (95% CI 6.4–9.4) for cabozantinib (Table 3; Fig. 1). PFS was longer for axitinib compared with everolimus: aHR was 0.63 (95% CI 0.45–0.88; *p* = 0.007); and estimated median PFS was 6.5 months (95% CI 4.6–7.8) for axitinib versus 3.7 months (95% CI 3.5–4.4) for everolimus (Table 3; Fig. 2).

#### Overall survival

##### Naïve indirect comparison

The naïve indirect comparative analysis showed OS was shorter for axitinib than cabozantinib: median OS was 15.2 months (95% CI 12.8–18.5) for axitinib versus 21.5 months (95% CI 17.1–not estimable) for cabozantinib (Table 4). OS between axitinib and everolimus: median OS was 15.2 months (95% CI 12.8–18.5) for axitinib versus 16.5 months (95% CI 13.3–19.1) for everolimus (Table 4).

**Table 4:** Overall survival – naïve and MAIC (base-case and sensitivity) analyses

	Naïve	Base-case analysis	Sensitivity analysis
Axitinib (AXIS) vs. cabozantinib (METEOR)			
Axitinib, median (95% CI) OS, months	15.2 (12.8–18.5)	21.5 (15.7–27.3)	15.7 (12.8–21.5)
Cabozantinib, median (95% CI) OS, months	21.5 (17.1–NE)	21.5 (17.1–NE)	21.5 (17.1–NE)
aHR (95% CI)	–	1.00 (0.69–1.46)	1.35 (0.95–1.92)
Axitinib (AXIS) vs. everolimus (METEOR)			
Axitinib, median (95% CI) OS, months	15.2 (12.8–18.5)	21.7 (13.5–28.3)	15.5 (12.8–23.1)
Everolimus, median (95% CI) OS, months	16.5 (13.3–19.1)	16.5 (13.3–19.1)	16.5 (13.3–19.1)
aHR (95% CI)	–	0.63 (0.42–0.96)	0.84 (0.59–1.18)

*aHR* adjusted hazard ratio, *CI* confidence interval, *MAIC* matching-adjusted indirect comparison, *MSKCC* Memorial Sloan Kettering Cancer Center, *NE* not estimable, *OS* overall survival

##### Matching-adjusted indirect comparison

**Fig. 3:**
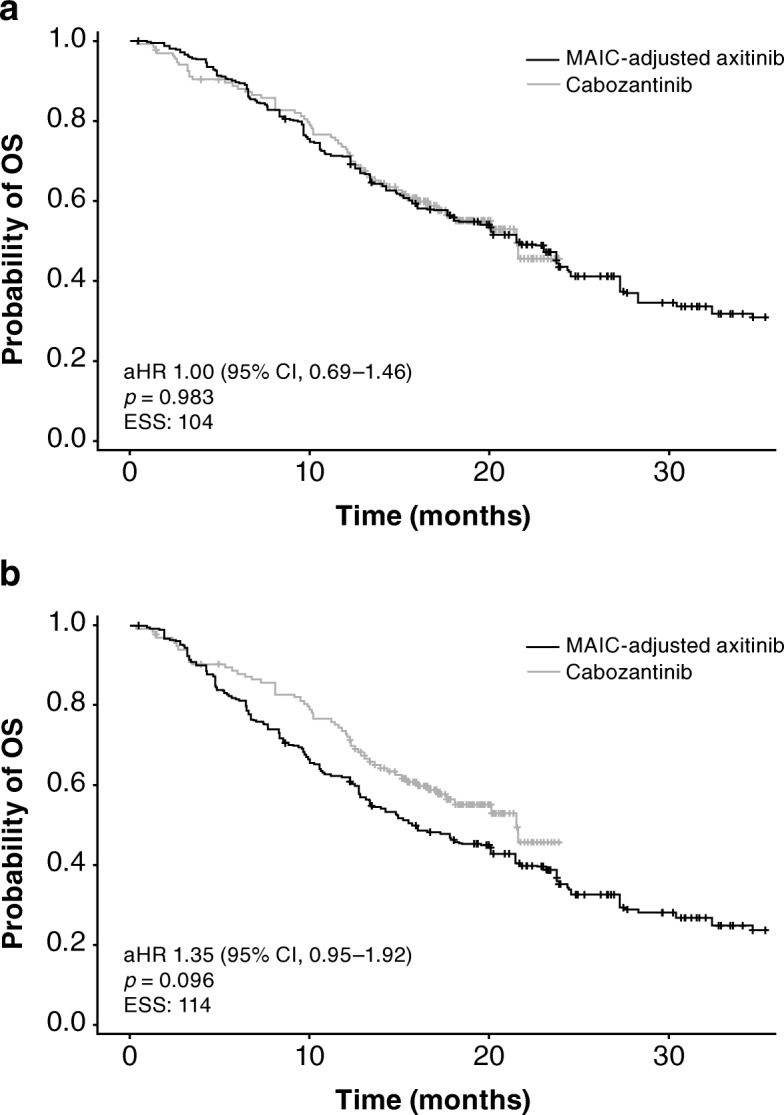
Kaplan–Meier curves for overall survival for axitinib versus cabozantinib: (**a**) base-case analysis; (**b**) sensitivity analysis. aHR, adjusted hazard ratio; CI, confidence interval; ESS, effective sample size; MAIC, matching-adjusted indirect treatment comparison; OS, overall survival

In the base-case analysis, there was no difference in OS between axitinib and cabozantinib: aHR = 1.00 (95% CI 0.69–1.46; *p* = 0.983); and estimated median OS was 21.5 months (95% CI 15.7–27.3) for axitinib versus 21.5 months (95% CI 17.1–not estimable) for cabozantinib (Table 4; Fig. 3). OS was significantly longer for axitinib than everolimus: aHR was 0.63 (95% CI 0.42–0.96; *p* = 0.032); and estimated median OS was 21.7 months (95% CI 13.5–28.3) for axitinib versus 16.5 months (95% CI 13.3–19.1) for everolimus (Table 4; Fig. 3).

**Fig. 4:**
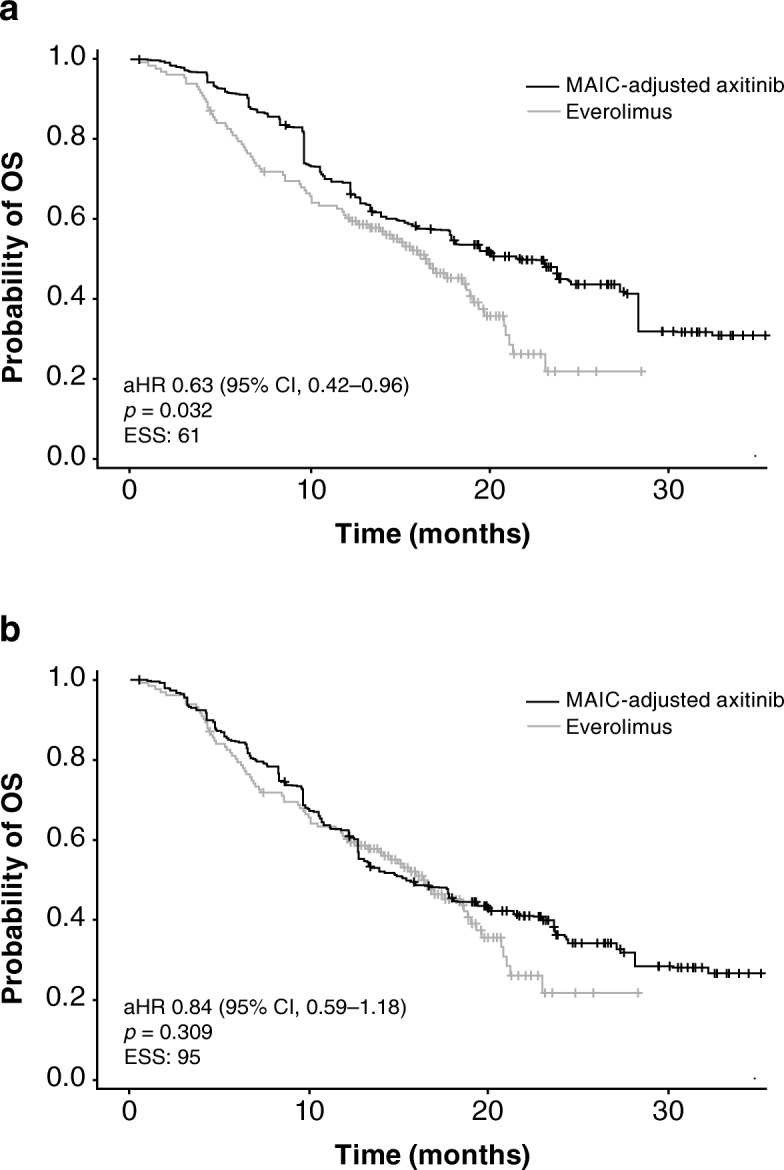
Kaplan–Meier curves for overall survival for axitinib versus everolimus: (**a**) base-case analysis; (**b**) sensitivity analysis. aHR, adjusted hazard ratio; CI, confidence interval; ESS, effective sample size; MAIC, matching-adjusted indirect treatment comparison; OS, overall survival

In the sensitivity analysis, OS was shorter for axitinib compared with cabozantinib: aHR was 1.35 (95% CI 0.95–1.92; *p* = 0.096); and estimated median OS was 15.7 months (95% CI 12.8–21.5) for axitinib versus 21.5 months (95% CI 17.1–not estimable) for cabozantinib (Table 4; Fig. 4). However, differences were not statistically significant. No difference was observed in OS between axitinib and everolimus in the sensitivity analysis: aHR was 0.84 (95% CI 0.59–1.18; *p* = 0.309); and estimated median OS was 15.5 months (95% CI 12.8–23.1) for axitinib versus 16.5 months (95% CI 13.3–19.1) for everolimus (Table 4; Fig. 4).

## Discussion

For patients with mRCC who progressed on first-line sunitinib, several treatment options with other targeted agents are available as second-line or subsequent therapies. However, evidence on their relative efficacy is limited due to a lack of head-to-head trials to guide the optimal choice of therapy. The current study was conducted with the aim of comparing PFS and OS for axitinib versus cabozantinib or everolimus in patients with mRCC who were previously treated with sunitinib. To date, the AXIS trial is the only head-to-head phase 3 trial that evaluated axitinib against a comparator, sorafenib, which was the standard of care in the second-line setting at the time of the trial [4]. Other phase 3 trials conducted in the second-line setting, such as RECORD-1 (everolimus vs. placebo) [8], METEOR (cabozantinib vs. everolimus) [6, 7], and CHECKMATE025 (nivolumab vs. everolimus) [6], used everolimus as a comparator. Since a standard mixed treatment comparison was not possible due to a disjointed network in the prior sunitinib subgroup, an MAIC comparison was necessary to determine the comparative efficacy between axitinib and everolimus, and between axitinib and cabozantinib.

Using the MAIC analyses [9], prior sunitinib-treated patient subgroups were compared across the AXIS and METEOR trials, adjusting for differences in baseline patient characteristics. In the base-case analysis, all patient characteristics, including MSKCC score derived in AXIS assuming ECOG PS 1 was a risk factor, were used as matching variables. In the sensitivity analysis, the same patient characteristics were used, except MSKCC score in AXIS was derived assuming ECOG PS 1 was not a risk factor. When comparing axitinib with everolimus, a statistically significant advantage in PFS for axitinib versus everolimus was observed in both the base-case (*p* = 0.002) and sensitivity (*p* = 0.007) analyses. The benefit of axitinib compared with everolimus was also seen for OS in the base-case analysis (*p* = 0.032), but not in the sensitivity analysis (*p* = 0.309). When comparing axitinib with cabozantinib, the base-case analysis suggested no difference in PFS (*p* = 0.423) or OS (*p* = 0.983) between the two treatments. The sensitivity analysis suggested a marginal benefit for cabozantinib versus axitinib for PFS (*p* = 0.050); however, the differences in OS did not reach statistical significance (*p* = 0.096). Different conclusions in the base-case and sensitivity analyses for comparison of axitinib with cabozantinib arise from differences in how the MSKCC score was calculated in AXIS in these two analyses. The disparities between the base-case and sensitivity analyses in this study underscore the importance of adjusting for the differences in baseline characteristics, and that the naïve indirect comparisons are potentially problematic and should be avoided.

The OS results in the current analyses should nevertheless be interpreted with caution due to the difference in maturity of the OS data across the trials. In addition, OS is impacted by study treatments and by post-study treatments. Indeed, a higher percentage of axitinib-treated patients in the AXIS trial received post-study therapies compared with everolimus- or cabozantinib-treated patients in the METEOR trial (60, 55, and 48%, respectively); this may be explained, in part, by the difference in the maturity of the trials. The most common post-study systemic treatments in axitinib-treated patients were everolimus (43%) and sorafenib (20%). In the METEOR trial, the most common post-study treatments were axitinib (39%), followed by either pazopanib (7%) or sorafenib (7%) in everolimus-treated patients; and everolimus (25%) and axitinib (18%) in cabozantinib-treated patients. The OS results would have been confounded by the observed imbalance in the composition of subsequent systemic therapies between the trials, which could not be corrected for, and, more importantly, the fact that 39% of everolimus-treated patients and 18% of cabozantinib-treated patients in the METEOR trial received post-study axitinib, and 43% of axitinib-treated patients in the AXIS trial received post-study everolimus (none received cabozantinib).

The study results are not in agreement with the findings of three studies that compared survival between axitinib, everolimus, and cabozantinib in the second-line setting using different analytical methods [22–24]. In an NMA study, Amzal et al. [23] reported HRs in favour of cabozantinib versus axitinib for PFS (2.13 [95% CI 1.32–3.43]) and OS (1.96 [95% CI 0.68–5.7]). Sherman et al. [24] conducted a weight-adjusted indirect comparison of prior sunitinib-treated patients with second-line mRCC treated with everolimus from the RECORD-1 trial and axitinib-treated patients from the AXIS trial, and found no statistically significant differences between axitinib and everolimus in PFS; however, the sample size was small (*n* = 43) and included patients who were sunitinib-intolerant. Similarly, in a retrospective chart review of the axitinib versus everolimus cohort study by Vogelzang et al. [22], no differences in OS and PFS were observed between axitinib and everolimus in the overall study population. However, subgroup analyses suggested a significant OS benefit with axitinib among patients who had received sunitinib or sorafenib as first-line treatment for < 6 months.

There are inherent limitations to our study. Although the clinical trial design, inclusion criteria, and outcomes definitions were comparable between the AXIS and METEOR trials, some differences were noted that could have potentially impacted the comparison. Firstly, differences in timing of PFS assessments by an independent review committee may have led to overestimation of PFS for everolimus and cabozantinib (METEOR: 8 weeks for the first 12 months, and every 12 weeks thereafter) compared with axitinib (AXIS: after 6 and 12 weeks of therapy, and every 8 weeks thereafter). Secondly, the difference between the MSKCC scores derived in the AXIS versus METEOR trial could not be fully adjusted for in our analyses. Thirdly, there was a potential for residual confounding due to omitting some patient characteristics from analyses (e.g., number of metastases, duration of the prior sunitinib treatment, and time since diagnosis), since these were not available for the prior sunitinib-treated patient subgroups in the METEOR trial. Although the current study adjusted for the main prognostic factors [25–27], the impact of excluding other characteristics from the analyses is unclear. Finally, differences in subsequent therapies, including the high percentages of everolimus- and cabozantinib-treated patients who received axitinib post-study in the METEOR trial, could not be accounted for when analysing the OS data.

Despite these limitations, the MAIC analysis offers advantages over a naïve comparison since it attempts to adjust for differences in baseline patient characteristics, and thus allows for a more comprehensive comparison of the treatment effects.

## Conclusions

Although assumptions were required, an indirect comparison using MAIC based on the AXIS and METEOR trials suggested no differences in PFS and OS in the base-case analysis between axitinib and cabozantinib in prior sunitinib-treated patients. Sensitivity analyses suggested a significant PFS gain with cabozantinib compared with axitinib; however, no significant difference in OS was observed. For axitinib versus everolimus, a beneficial treatment effect was observed for PFS, and potentially for OS, in patients with mRCC previously treated with sunitinib. Disparities in the base-case and sensitivity analyses between axitinib and cabozantinib highlight the importance of adjusting for differences in trial populations for indirect treatment comparisons. Additionally, these analyses demonstrate that MAIC can improve the reliability of indirect comparisons compared with using aggregate data alone; however, a randomised, head-to-head, controlled trial is needed if definitive conclusions are to be made.

## Abbreviations

aHR: adjusted hazard ratio
CI: confidence interval
ECOG PS: Eastern Cooperative Oncology Group performance status
ESS: effective sample size
KPS: Karnofsky performance status
MAIC: matching-adjusted indirect comparison
mRCC: metastatic renal cell carcinoma
MSKCC: Memorial Sloan Kettering Cancer Center
NMA: network meta-analysis
OS: overall survival
PD-1: programmed cell death-1
PD-L1: programmed cell death ligand-1
PFS: progression-free survival
RCC: renal cell carcinoma
TKI: tyrosine kinase inhibitor
VEGFR: vascular endothelial growth factor receptor
